# Activity Prediction and Molecular Mechanism of Bovine Blood Derived Angiotensin I-Converting Enzyme Inhibitory Peptides

**DOI:** 10.1371/journal.pone.0119598

**Published:** 2015-03-13

**Authors:** Ting Zhang, Shaoping Nie, Boqun Liu, Yiding Yu, Yan Zhang, Jingbo Liu

**Affiliations:** 1 Laboratory of Nutrition and Functional Food, Jilin University, Changchun, Jilin, China; 2 State Key Laboratory of Food Science and Technology, Nanchang University, Nanchang, Jiangxi, China; Escola Paulista de Medicina, BRAZIL

## Abstract

Development of angiotensin I-converting enzyme (ACE, EC 3.4.15.1) inhibitory peptides from food protein is under extensive research as alternative for the prevention of hypertension. However, it is difficult to identify peptides released from food sources. To accelerate the progress of peptide identification, a three layer back propagation neural network model was established to predict the ACE-inhibitory activity of pentapeptides derived from bovine hemoglobin by simulated enzyme digestion. The pentapeptide WTQRF has the best predicted value with experimental IC_50_ 23.93 μM. The potential molecular mechanism of the WTQRF / ACE interaction was investigated by flexible docking.

## Introduction

Hypertension is a risk factor for cardiovascular diseases including coronary heart disease, peripheral artery disease and stroke. [1, 2] Recently, several food-derived bioactive peptides have been found playing a significant role in decreasing blood pressure. Therefore, more and more attention has been paid to peptides from food sources with antihypertensive activity. [3]

Most of the antihypertension peptides regulate blood pressure by inhibiting the activity of angiotensin I-converting enzyme (ACE, EC.3.4.15.1). ACE is a zinc- and chloride- dependent metallopeptidase, which belongs to the M2 family of zinc metallopeptidases. [4, 5] It converts angiotensin I to angiotensin II (a potent vasoconstrictor) as well as inactivates the vasodilator bradykinin. [6] ACE plays a crucial role in the renin-angiotensin system (RAS), which is well known for its regulation of blood pressure and fluid homeostasis. [7, 8] Nowadays, inhibitors of ACE have been considered as first-line therapy for hypertension. [9, 10] It has been reported that a number of bioactive peptides, which derived from food sources, have ACE-inhibitory activity. Chibuike C. Udenigwe [11] summarized the major approaches in bioactive peptides research as the classical approach, the bioinformatics approach and the integrated approach. This classification is also suitable for ACE-inhibitory peptides.

The classic approach is the most widely used method for the discovery of ACE-inhibitory peptides from food proteins, involving peptides production (solvent extraction, enzyme hydrolysis, and microbial fermentation), purification (membrane-based separation and chromatography techniques) and identification (mass spectrometry methods). There are a number of ACE-inhibitory peptides derived from different food sources and obtained by the classic approach. For instance, ACE-inhibitory peptides derived from soy protein such as DLP, DG, IA, ILAGNQ, FFL, IYLL, VMDKPQG, IFL, WL, TPRVF, YVVFK, PNNKPFQ, EDENNPFYLR, NWGPLV, IPPGVPYWT, VLIVP, LAIPVNKP, LPHF, SPYP and WL, were found in published articles. [12–17] In wheat germ hydrolysates, 16 peptides [18] with the IC_50_ value of less than 20 μM, composed of 2–7 amino acid residues were identified. And IAP [19] was identified in wheat gliadin hydrolysates. In pork meat hydrolysates digested by *in vitro* gastrointestinal digestion, 12 peptides were identified. [20] Also, in beef rump (biceps femoris) hydrolysates, Jang and Lee [21] identified VLAQYK.

In order to circumvent some challenges of the classical approach, the bioinformatics approach has been recently applied towards the discovery of ACE-inhibitory peptides encrypted in food proteins. This approach was recently used to study the distribution of ACE-inhibitory peptides within the primary structure of typical food proteins. [22]

Following the identification of bioactive peptides from protein sets by bioinformatics in databases populated following the classical approach, the remainder of the purportedly “inactive” peptides can be analyzed in silico to identify structural patterns that have previously been associated with known bioactivities. [11]

Moreover, the strengths of each approach can be combined as deemed fit to enhance the discovery and use of ACE-inhibitory peptides. Bioinformatics software can be used to simulate proteolytic specificities of enzymes in order to establish the peptide database in silico. [11] Quantitative structure-activity relationship (QSAR) studies are widely undertaken for modeling the bioactivities such as the bioactivity of ACE-inhibitory peptides [22–26] and the sweetness of compounds [27]. Neural network, as a kind of artificial intelligence, has been applied to modeling non-linear systems, simulating the chaos bioprocess and predicting the results. It turns out to have higher modeling accuracy and generalization capacity [28] and becomes a potentially effective tool in modeling the QSAR.

Bovine blood, as a by-product generated in great volume in industrial abattoirs, gives rise to several possibilities for their recovery and use. The use of bovine blood as a food component has been widely reported due in part to their high nutritional value and there are many studies have recently demonstrated that bovine blood proteins can be used to obtain bioactive peptides. This is important because it gives an added value to bovine blood. A number of bioactive peptides released from bovine hemoglobin hydrolysates have been reported. [29–32] It is a highly desirable but difficult task to identify bovine blood derived peptides. In this aspect, prediction model would be a useful technique to highlight potential ACE-inhibitory peptides identification.

It was found that ACE has two homologous domains (the N-domain and the C-domain), each containing an active center. [33] The C-domain of ACE has been proved to be the dominant angiotensin-I converting site, which has a conserved HEXXH zinc-binding motif, for controlling blood pressure and serving cardiovascular functions. [34, 35] If inhibitory peptides occupied the active site of the C-domain of ACE and bound to specific amino acid residues, ACE will lose its activity. Therefore, it is possible to reveal the ACE inactivation mechanisms by analyzing the structural consequences of ACE-inhibitor interactions. [34]

In this study, we established a database of potential pentapeptides derived from bovine hemoglobin by simulated enzyme digestion, and a prediction model of ACE-inhibitory pentapeptides by back propagation neural network (BPNN). The peptide with best predictive value was synthesized and its IC_50_ of ACE was measured. We also sought to elucidate the potential molecular mechanism of how the peptide with best predictive value exerts its ACE-inhibitory effects by automated molecular docking.

## Materials and Methods

### Materials and Chemicals

Angiotensin converting enzyme from rabbit lung, hippuryl-L-histidyl-L-leucine, hippuric acid and HPLC grade acetonitrile, trifluoroacetic acid were purchased from Sigma Chemical Co. (St. Louis, Mo., U.S.A.). All the other reagents were analytical grade.

### Dataset

A total of 24 unique pentapeptides were collected from published articles with IC_50_ of ACE from 0.00948μM to 848 μM in Table 1. [16, 36–48] Due to the wide range of IC_50_ value, the negative logarithm of IC_50_ (pIC_50_) was used ranging from-2.928 to 2.023.

**Table 1:** The IC_50_ (μM) of ACE of 24 pentapeptides as taken from literature.

No.	Sequence	IC_50_	No.	Sequence	IC_50_
1	ARHPH	0.0156	13	MRWRD	2.1
2	DIGYY	3.4	14	RINKK	0.0183
3	DKIHP	113.1	15	RYLGY	0.71
4	DYVGN	0.72	16	SLPQN	0.00948
5	ERYPI	8.76	17	TVVPG	2.2
6	EVPKA	324.77	18	TYKEE	0.0186
7	IKYGD	4.5	19	TYLGS	0.86
8	KDERF	848	20	VKQGF	20.3
9	KDYRL	26.5	21	VLIVP	1.69
10	LDIQK	27.6	22	WVPSV	0.501
11	LPYPY	28.9	23	YTAGV	23.38
12	LVQGS	43.7	24	YVVFK	44

### Molecular Descriptors Calculation

Ten molecular descriptors were calculated for analogs of peptides including structural, spatial, thermodynamic and electronic. Structural descriptors included the number of rotatable bond (Rotbond), the number of hydrogen bond acceptor (H bond acceptor), the number of hydrogen bond donor (H bond donor), the number of ring (Ring) and the number of arcomatic ring (AR). [49–51] The molecular fractional polar surface area (PSA) [52] was used as spatial descriptor. The thermodynamic descriptors were taken to describe the hydrophobic character (ALog P: logarithm of the partition coefficient in octanol / water and Log D: logarithm of the partition coefficient in octanol / water in pH 7.4) and refractivity (MR: molar refractivity). [49] The atomic polarizabilities (Apol) were calculated as electronic descriptors. [50] Peptide structures were generated by Accelrys Discovery Studio 3.5 software (Accelrys Inc., San Diego, USA) and the energy was minimized with CHARMm program using steepest descent and conjugate gradient techniques. All descriptors were calculated by calculate molecular properties protocol.

### Modeling by Back-Propagation Neural Network (BPNN)

To construct a BPNN, the Levenberg-Marquardt algorithm [53] was used to train the network. With all data normalized, the descriptors and the pIC_50_ of dataset were introduced as input and output values, respectively. In order to avoid overfitting, the dataset was randomly classified for training, validation, and test sets. To verify the suitable number of nodes in hidden layer and transfer function, the mean square error (MSE) and pearson correlation coefficient (R) of each BPNN model were calculated.

### Simulated Enzyme Digestion and BPNN Prediction

Pepsin and trypsin degrade food proteins into peptides, which are critical in human digestion of protein. The online software Peptide Cutter (http://web.expasy.org/peptide_cutter/) can predict potential cleavage sites cleaved by proteases, including pepsin and trypsin, in a certain protein sequence. As hemoglobin is composed of four poly peptide subunits, two α and two β, we used Peptide Cutter to predict the potential pentapeptides of subunit α (accession number, P01966) and β (accession number, P02070), respectively. All pentapeptides were described by descriptors mentioned above. And then, put the normalized descriptors into the best BPNN model as input data to predict the ACE-inhibitory activity.

### Peptide Synthesis

The peptide was synthesized (ChinaPeptides Co. Ltd., Shanghai, China) by the solid phase procedure peptide using FMOC protected amino acids synthesis. The purity (95%) and the molecular masses of the peptides were determined by HPLC and mass spectrometry.

### Assay for ACE-Inhibitory Activity

The ACE-inhibitory activity of the peptide with best predict value was measured according to Liu, et al. [54]

### Docking

The 3-D structure of human ACE was imported from Protein Data Bank (1O8A.pdb, a crystal structure of human ACE). The structure of peptide was generated by DS and the energy was minimized with CHARMm program. Automated molecular docking was performed using the flexible docking tool of DS software in the presence of cofactors (zinc and chloride ions). The binding site with a radius of 15 Å and a coordinates x: 45.0463, y: 38.6842, z: 45.8268. Evaluation of the molecular docking was performed according to the scores and total potential energy in order to obtain the best pose of peptide. The DS software was used to identify the hydrogen bonds as well as the hydrophobic, hydrophilic, electrostatic and coordination interactions between residues present within ACE active site.

## Results and Discussion

### Structural Features Analysis

Most of the ACE-inhibitory peptides derived from egg white hydrolysates in our lab previously [55–57] were pentapeptides. Therefore, we are interested in ACE-inhibitory pentapeptide and try to build a prediction model based on a pentapeptides database (Table 1). It shows that 75% of the pentapeptides taken from literature have 1 or 2 hydrophobic amino acid residues. A half of these pentapeptides contain aromatic or hydrophobic amino acid residues at the C-terminus, which are beneficial to ACE-inhibitory activity. [24] In addition, 66.67% of these pentapeptides contain hydrophilic amino acid residues in the second amino acid residues from the C-terminus. The aforementioned common structural features can be used to instruct ACE-inhibitory peptides’ design.

The molecular descriptor (Tables 2, 3) calculation is a logic and mathematical procedure which converts the chemical information of a molecule into some useful data. Most of the number ranges of descriptors of Pentapeptides derived from hemoglobin simulated enzyme digestion included in the scope of the reported pentapeptides, except the molar refractivity of GHGAK. The range of MR of reported pentapeptides is 111.291 to 186.874, while the GHGAK is 107.89. Nevertheless, the predicted IC_50_ of GHGAK is the fourth lowest among the seven hemoglobin pentapeptides, because the BPNN is a nonlinearity model, leading to a nonlinear relationship between the input and the output.

**Table 2:** Descriptors of 24 peptides.

No.	ALog P	MR	Apol	Log D	Rot bond	Ring	AR	H bond acceptor	H bond donor	PSA
1	-3.40648	150.01	21,842.80	-5.211	16	3	2	8	9	0.472
2	-0.5315	148.186	23,371.50	-3.62	17	2	2	10	7	0.417
3	-1.57883	140.478	21,007.90	-6.343	18	2	1	9	6	0.43
4	0.1427	125.13	20,031.40	-6.645	16	1	1	10	7	0.496
5	-4.17411	162.498	24,053.20	-4.59	19	2	1	9	8	0.43
6	-1.75544	123.385	18,340.80	-6.673	16	1	0	8	5	0.423
7	1.17668	137.363	21,169.30	-5.804	19	1	1	9	7	0.438
8	-7.64934	157.324	24,572.40	-9.119	24	1	1	10	9	0.492
9	-0.97576	165.622	24,679.50	-6.381	23	1	1	9	10	0.46
10	-1.4409	141.829	20,908.70	-6.554	22	0	0	9	7	0.444
11	-0.18578	165.819	24,392.10	-0.486	13	4	2	8	5	0.32
12	2.40026	116.474	16,937.50	-5.206	16	0	0	8	7	0.459
13	-1.4177	186.874	28,111.80	-5.953	24	2	2	9	12	0.502
14	4.6319	160.264	22,613.60	-7.87	25	0	0	7	11	0.482
15	1.46736	169.636	25,104.30	-2.379	19	2	2	8	10	0.419
16	0.616643	127.519	18,710.70	-6.491	16	1	0	9	7	0.486
17	1.08715	111.291	15,909.40	-3.97	11	1	0	7	5	0.392
18	-6.51692	146.046	23,291.50	-8.625	22	1	1	12	8	0.483
19	2.27823	127.141	19,082.70	-4.319	15	1	1	9	8	0.439
20	0.263857	141.365	20,960.90	-5.071	19	1	1	7	7	0.418
21	-0.2278	137.092	18,644.10	-0.405	14	1	0	6	4	0.299
22	-10.8299	147.072	21,425.80	-1.896	13	3	2	7	6	0.354
23	-0.94115	120.919	18,224.60	-3.266	13	1	1	8	7	0.424
24	-1.6435	168.555	24,814.30	-0.956	19	2	2	7	7	0.34

**Table 3:** Pentapeptides derived from hemoglobin simulated enzyme digestion.

	AAWGK	AHRYH	FTPVL	GHGAK	PTTKT	TSKYR	WTQRF
Proteases	Trypsin	Pepsin	Pepsin	Trypsin	Pepsin	Pepsin	Pepsin
Subunit	α	β	β	α	α	α	β
ALogP	-4.704	-5.548	-1.547	-7.589	-7.283	-6.927	-3.789
MR	130.843	168.635	145.081	107.89	125.207	157.139	186.729
Apol	19,654.10	25,184.50	20,818.60	16,291.60	18,110.90	23,078.30	27,793.80
LogD	-4.702	-4.691	-1.54	-6.806	-7.28	-6.915	-2.911
Rot bond	15	19	14	15	16	21	21
Ring	2	3	2	1	1	1	3
AR	2	3	1	1	0	1	3
H bond acceptor	6	9	7	7	9	9	8
H bond donor	7	11	5	7	9	12	11
PSA	0.414	0.47	0.326	0.487	0.46	0.492	0.439
BPNN output	0.5093	0.7990	0.4534	0.4983	0.2890	0.3204	0.8501
Predicted IC_50_ (μM)	2.5513	0.0938	4.8240	2.8916	31.4255	21.9784	0.0524

### BPNN model Building

**Fig 1:**
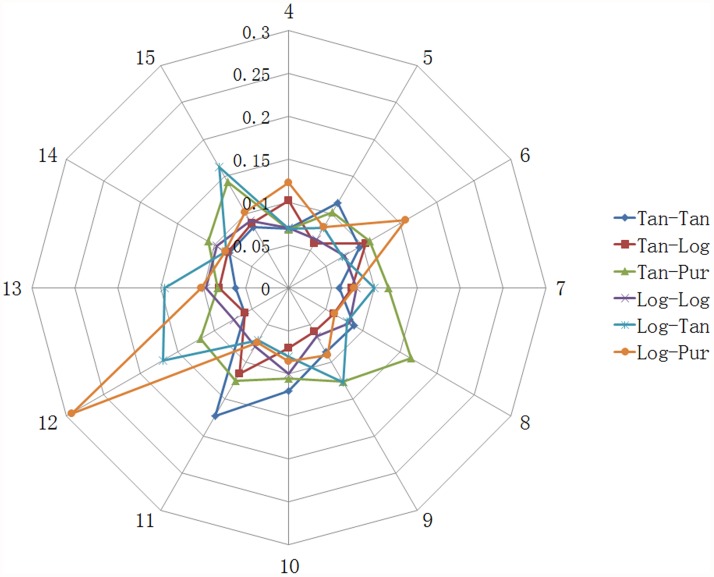
The MSE of different structure of BPNN models.

**Fig 2:**
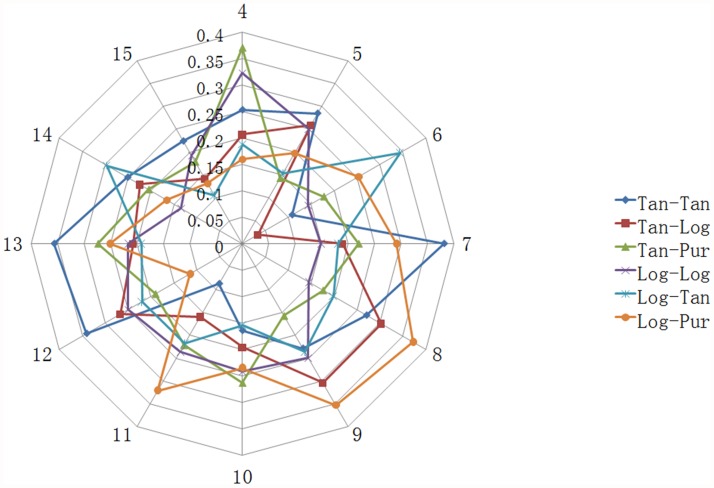
The coefficient of determination (square of pearson correlation coefficient) of different structure of BPNN models.

**Fig 3:**
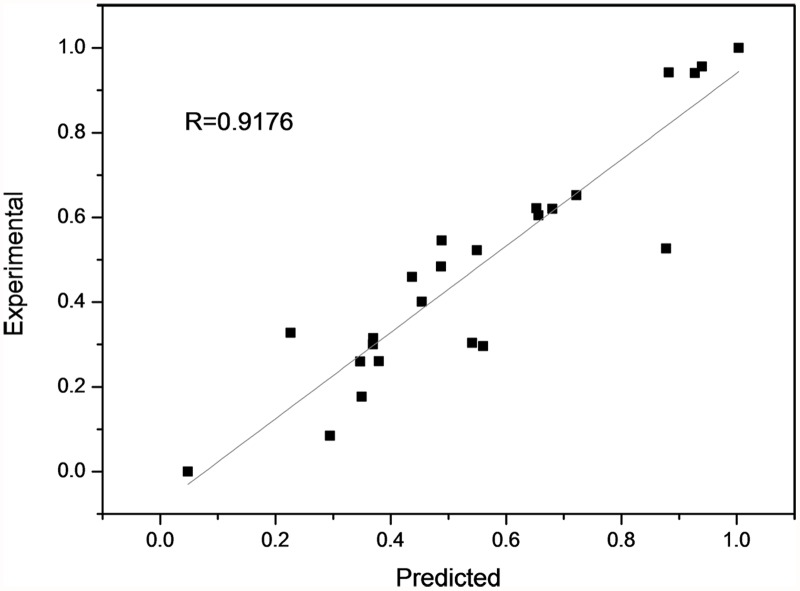
Predicted versus experimental plots for BPNN model.

There were three layers in the BPNN model (input layer, hidden layer and output layer), while the number of nodes in hidden layer was not certain. So that we trained BPNN models with different number of nodes in hidden layer and different transfer functions to obtain the suitable model structure (Fig. 1, Fig. 2 and S1 Table, S2 Table). For the log-sigmoid & purelin transfer function, the highest MSE appears when the number of hidden layers is twelve. However, in terms of the tan-sigmoid & tan-sigmoid transfer function, the MSE is relatively low when the number of hidden layers is between four and fifteen, and the lowest MSE (0.0587 ± 0.0351) is obtained when it reaches seven. Besides, seven hidden layers with the log-sigmoid & purelin transfer function bring the highest determination coefficient (square of R, 0.3819 ± 0.2781). Therefore, we decided to select 10–7–1 as the topological structure of BPNN and the tan-sigmoid transfer function as the transfer function between both input layer & hidden layer and hidden & output layer. To improve the accuracy of the final model, we set the aim as R > 0.9 and trained 10–7–1 BPNN with tan-sigmoid transfer function & tan-sigmoid transfer function several times. The MSE and correlation coefficients of this model were in acceptable ranges as shown in Table 4. These correlation coefficients indicate that there is a strong correlation between the predicted and experimental result and the MSE (0.162) is acceptable. The plots of experimental versus predicted values (Fig. 3) confirmed the discussed results. Hence, we chose this one as the final model. To our knowledge, it was the first time applying BPNN to predict the IC_50_ of ACE of pentapeptide. In our previous work [58], a BPNN model was built to predict the IC_50_ of ACE of tripeptide. The MSE of the tripeptide model (0.2148) is higher than this pentapeptide model (0.0162). Meanwhile, the R of the tripeptide model (0.854) was less than this new model (0.9176). These differences possibly resulted from the difference of descriptors.

**Table 4:** MSE and correlation coefficients of final BPNN model.

MSE	Pearson	Kendall	Spearman
0.0162	0.9176**	0.7536**	0.8887**

** Significant difference is p<0.01.

### Activity Prediction and Experimental Verification

After simulated enzyme digestion of hemoglobin, seven pentapeptides among all the cleavage fragments were obtained. The descriptor calculated value and the predicted IC_50_ were shown in Table 3. The pentapeptide WTQRF and AAWGK contain two or three hydrophobic amino acid residues, which is in agreement with the common features of the 24 reported pentapeptides, and show a low predicted IC_50_. The AHRYH, WTQRF and FTPVL contain hydrophpbic or aromatic amino acid residues in the C- terminus which contribute to ACE inhibition. For the second amino acid residues from C-terminus, the PTTKT, WTQRF and AAWGK contain hydrophilic amino acid residues, which also match the common features of the 24 reported pentapeptides. Obviously, WTQRF conforms to all the aforementioned common features. It include three hydrophilic amino acid residues (Thr, Gln, Arg) and two hydrophobic amino acid residues with aromatic (Trp) / heterocyclic (phe) ring in their side chain. It molecular weight is 736.83 g / mol. The hydrophobic amino acid residues in both of the N- and C- terminus as well as the hydrophilic amino acid residues in the second amino acid residues from C-terminus may make main contribution to ACE inhibition. The result of predicted IC_50_ indicated that the peptide WTQRF has the best inhibitory activity of ACE. The experimental IC_50_ of WTQRF is 23.93 μM, which is similar to a pentapeptide identified from egg white protein in our lab before (RVPSL, IC_50_ 20 μM). [54] The difference between predicted and experimental IC50 may bring from data normalization.

### Molecular Docking

**Fig 4:**
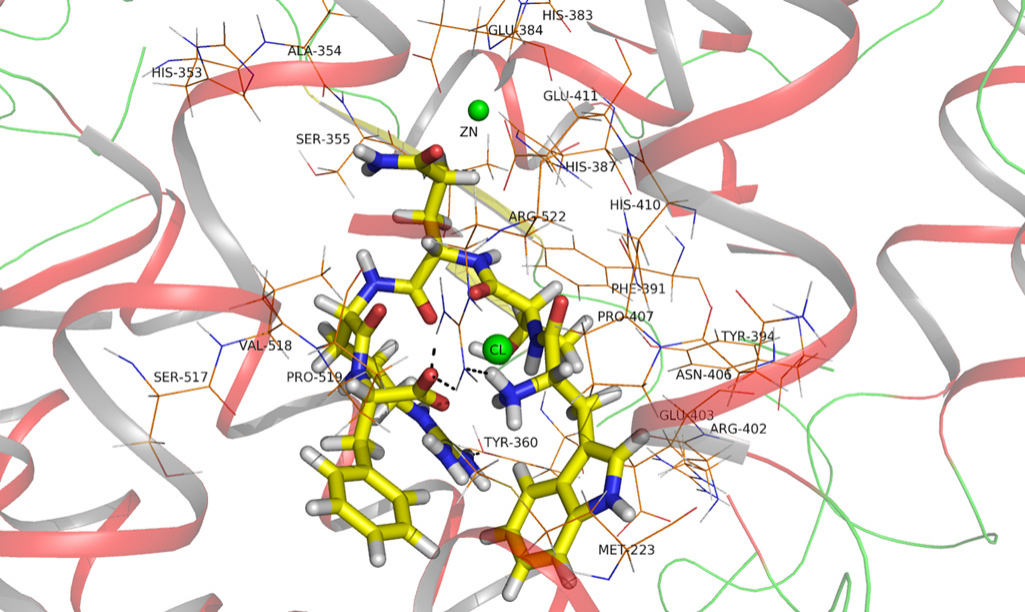
WTQRF binding with the active site of ACE, the conformation extracted from docking result.

Interactions between ACE and inhibitor are fundamental to ACE-inhibitory processes. In this work, docking is used to predict the preferred orientation of WTQRF to ACE when they bound to each other to form a stable complex. The free energy of binding represents the binding affinity between ACE and WTQRF. The molecular docking study of WTQRF presenting within the ACE catalytic site in the presence of the cofactor showed the best pose with potential binding energy:- 749.747 kj / mol. It is indicated that the ligand bind tightly to the receptor and the ACE-WTQRF complex is stable. As we know, ligand-protein affinities are influenced by non-covalent intermolecular interactions between the two molecules. The best pose of WTQRF was stabilized mainly by hydrophobic and hydrophilic interactions (Fig. 4), electrostatic interactions (Electrostatic energy:- 154.728 kj / mol; Van der Waals energy:- 898.743 kj / mol) and hydrogen bond (Table 5).

**Table 5:** Hydrogen bonds observed between ACE and the best docking pose of WTQRF.

Donor-Acceptor	ARG522:HH12—WTQRF:O53	ARG522:HH22—WTQRF:O53	WTQRF:H54—ARG522:NH2	WTQRF:H92—TYR360:OH
Distance (Å)	1.36	1.86	2.44	1.76

The hydroxyl group of Tyr 360, the guanidine and the ɛ-amino group of Arg 522 showed significant importance of the binding between WTQRF and ACE by hydrogen bond. The docking result suggested that WTQRF contact with residues Met 223, Ala 354, 356, Tyr 360, 394, Phe 391, Pro 407, 519, Val 518 by hydrophobic interactions and with residues His 353, 383, 387, 413, Ser 355, 517, Arg 402, 522, Glu 384, 403, 411, Asn 406, Val 518 by hydrophilic interactions. The active site of ACE was constituted of a zinc ion and a HEXXH…E motif, including His 383, Glu 384, His 387 on helix α 13 and Glu 411 on helix α 14. [34, 59] The WTQRF were positioned to interact with the HEXXH…E motif. Furthermore, the interactions between inhibitors and Zn^2+^ at the ACE active site also play a significant role in modulating catalysis. [60] It is believed that the shorter distance between the Zn^2+^ and the carbonyl oxygen of the peptide, the greater the degree of ACE inhibition. [34]

## Conclusion

In this present work, a back propagation neural network model was built to predict the IC_50_ of ACE of pentapeptides. The topological structure was 10–7–1 and the transfer function was tan-sigmoid transfer function & tan-sigmoid transfer function. We also built a database of potential pentapeptides derived from bovine hemoglobin by simulated enzyme digestion and found that WTQRF has the highest predictive value with experimental IC_50_ 23.93 μM. The molecular docking result indicated that Tyr 360 and Arg 522 gave a significantly contribution to the stabilization between WTQRF and ACE. The result also demonstrated that the short distance between the Zn^2+^ and the carbonyl oxygen of the peptide are desirable to the ACE-inhibitory activity. The enzyme kinetics investigation as well as docking and molecular dynamics simulation analysis will be our further study.

## Supporting Information

S1 Table The MSE of different structure of BPNN model. (DOC)

S2 Table The coefficient of determination (square of pearson correlation coefficient) of different structure of BPNN models. (DOC)
