# Supplementary material for: Activity Prediction and Molecular Mechanism of Bovine Blood Derived Angiotensin I-Converting Enzyme Inhibitory Peptides
Source: PLoS One. 2015 Mar 13;10(3):e0119598. doi: 10.1371/journal.pone.0119598 (PMC4358945; doi:10.1371/journal.pone.0119598)
Supplement: S1 Table — (DOC) [file pone.0119598.s001.doc]

**S1_Table** The MSE of different structure of BPNN models

| No. | Tan | Tan | Tan | Log | Log | Log |
| --- | --- | --- | --- | --- | --- | --- |
|  | Tan | Log | Pur | Log | Tan | Pur |
| 4 | 0.0689 ± 0.0416 | 0.1017 ± 0.0994 | 0.0677 ± 0.0159 | 0.0704 ± 0.0372 | 0.0686 ± 0.0261 | 0.1229 ± 0.0511 |
| 5 | 0.1141 ± 0.1356 | 0.0598 ± 0.0191 | 0.1013 ± 0.0246 | 0.0648 ± 0.0267 | 0.0810 ± 0.0282 | 0.0816 ± 0.0266 |
| 6 | 0.0949 ± 0.0283 | 0.1037 ± 0.0338 | 0.1096 ± 0.0586 | 0.0751 ± 0.0190 | 0.0721 ± 0.0608 | 0.1575 ± 0.2050 |
| 7 | 0.0587 ± 0.0351 | 0.0734 ± 0.0213 | 0.1159 ± 0.0612 | 0.0794 ± 0.0274 | 0.0997 ± 0.0566 | 0.0765 ± 0.0375 |
| 8 | 0.0878 ± 0.0576 | 0.0604 ± 0.0147 | 0.1650 ± 0.0911 | 0.0819 ± 0.0101 | 0.0789 ± 0.0246 | 0.0612 ± 0.0406 |
| 9 | 0.0868 ± 0.0486 | 0.0590 ± 0.0158 | 0.1267 ± 0.0642 | 0.0658 ± 0.0181 | 0.1271 ± 0.1074 | 0.0904 ± 0.0569 |
| 10 | 0.1202 ± 0.0934 | 0.0707 ± 0.0296 | 0.1060 ± 0.0669 | 0.1006 ± 0.0901 | 0.0805 ± 0.0173 | 0.0862 ± 0.0378 |
| 11 | 0.1725 ± 0.1410 | 0.1157 ± 0.0830 | 0.1254 ± 0.0728 | 0.0789 ± 0.0410 | 0.0709 ± 0.0304 | 0.0739 ± 0.0092 |
| 12 | 0.0597 ± 0.0257 | 0.0588 ± 0.0156 | 0.1193 ± 0.0506 | 0.0743 ± 0.0242 | 0.1694 ± 0.1621 | 0.2933 ± 0.3834 |
| 13 | 0.0626 ± 0.0273 | 0.0813 ± 0.0448 | 0.0832 ± 0.0398 | 0.0968 ± 0.0490 | 0.1448 ± 0.1325 | 0.1019 ± 0.0586 |
| 14 | 0.0807 ± 0.0449 | 0.0828 ± 0.0414 | 0.1091 ± 0.0667 | 0.0971 ± 0.0397 | 0.0849 ± 0.0530 | 0.0859 ± 0.0186 |
| 15 | 0.0818 ± 0.0287 | 0.0870 ± 0.0343 | 0.1430 ± 0.1486 | 0.0899 ± 0.0361 | 0.1630 ± 0.1379 | 0.1019 ± 0.0325 |

The transfer function of each row is applied in input layer to hidden layer and hidden layer to output layer, respectively. Tan: tan-sigmoid transfer function; Log: log-sigmoid transfer function; Pur: liner transfer function. The results are presented as mean ± standard deviation (n=5) of pentaplicate determinations.
