# Supplementary material for: Activity Prediction and Molecular Mechanism of Bovine Blood Derived Angiotensin I-Converting Enzyme Inhibitory Peptides
Source: PLoS One. 2015 Mar 13;10(3):e0119598. doi: 10.1371/journal.pone.0119598 (PMC4358945; doi:10.1371/journal.pone.0119598)
Supplement: S2 Table — (DOC) [file pone.0119598.s002.doc]

**S2_Table** The coefficient of determination (square of pearson correlation coefficient) of different structure of BPNN models

| No. | Tan | Tan | Tan | Log | Log | Log |
| --- | --- | --- | --- | --- | --- | --- |
|  | Tan | Log | Pur | Log | Tan | Pur |
| 4 | 0.2531 ± 0.2712 | 0.2064 ± 0.1548 | 0.3702 ± 0.1264 | 0.3226 ± 0.2316 | 0.1880 ± 0.2770 | 0.1600 ± 0.2776 |
| 5 | 0.2846 ± 0.1733 | 0.2588 ± 0.2330 | 0.1432 ± 0.1176 | 0.2510 ± 0.2278 | 0.1537 ± 0.1673 | 0.1975 ± 0.1876 |
| 6 | 0.1086 ± 0.1354 | 0.0336 ± 0.0427 | 0.1781 ± 0.1488 | 0.1440 ± 0.1453 | 0.3442 ± 0.3737 | 0.2529 ± 0.3377 |
| 7 | 0.3819 ± 0.2781 | 0.1895 ± 0.1956 | 0.2202 ± 0.3019 | 0.1488 ± 0.1518 | 0.1812 ± 0.1988 | 0.2914 ± 0.2620 |
| 8 | 0.2713 ± 0.1713 | 0.3026 ± 0.1630 | 0.1763 ± 0.1795 | 0.1453 ± 0.0769 | 0.1982 ± 0.1260 | 0.3726 ± 0.2577 |
| 9 | 0.2290 ± 0.1616 | 0.3040 ± 0.1790 | 0.1577 ± 0.0917 | 0.2501 ± 0.1970 | 0.2365 ± 0.2360 | 0.3531 ± 0.3210 |
| 10 | 0.1647 ± 0.1447 | 0.1967 ± 0.1915 | 0.2632 ± 0.2146 | 0.2421 ± 0.1684 | 0.1545 ± 0.1439 | 0.2353 ± 0.2434 |
| 11 | 0.0877 ± 0.0970 | 0.1596 ± 0.2038 | 0.2217 ± 0.2419 | 0.2358 ± 0.2074 | 0.2180 ± 0.1678 | 0.3210 ± 0.0805 |
| 12 | 0.3564 ± 0.2948 | 0.3666 ± 0.1969 | 0.1902 ± 0.1301 | 0.2495 ± 0.2135 | 0.2197 ± 0.1830 | 0.1145 ± 0.1886 |
| 13 | 0.3564 ± 0.2275 | 0.2071 ± 0.2411 | 0.2734 ± 0.1222 | 0.2158 ± 0.1526 | 0.1917 ± 0.2438 | 0.2503 ± 0.1734 |
| 14 | 0.2508 ± 0.2921 | 0.2239 ± 0.1618 | 0.2047 ± 0.0517 | 0.1337 ± 0.1279 | 0.2965 ± 0.2187 | 0.1658 ± 0.1273 |

The transfer function of each row is applied in input layer to hidden layer and hidden layer to output layer, respectively. Tan: tan-sigmoid transfer function; Log: log-sigmoid transfer function; Pur: liner transfer function. The results are presented as mean ± standard deviation (n=5) of pentaplicate determinations.
